# Improvement of Subjective Olfactory Dysfunction in Chronic Rhinosinusitis With Nasal Polyps After Endoscopic Sinus Surgery

**DOI:** 10.3389/fsurg.2022.870682

**Published:** 2022-06-15

**Authors:** Ping Ye, Shaojuan He, Shuangmei Tang, Xinyu Xie, Chen Duan, Liqiang Zhang, John W. Steinke, Larry Borish, Xuezhong Li, Xin Feng

**Affiliations:** ^1^ Department of Otorhinolaryngology, Qilu Hospital of Shandong University, National Health Commission Key Laboratory of Otorhinolaryngology (Shandong University), Jinan, China; ^2^ Department of Medicine, University of Virginia Health System, Charlottesville, VA, United States; ^3^ Department of Microbiology, University of Virginia Health System, Charlottesville, VA, United States

**Keywords:** chronic rhinosinusitis with nasal polyps (CRSwNP), olfaction, endoscopic sinus surgery (ESS), prediction, eosinophil (EOS).

## Abstract

**Objective:**

Olfactory impairment is a common complaint in patients with chronic rhinosinusitis with nasal polyps (CRSwNP), but the influence of endoscopic sinus surgery (ESS) on olfaction and the factors predicting olfactory impairment are not fully understood. This study aimed to assess the effect of ESS on improving olfactory dysfunction in patients with CRSwNP and to identify factors that predict prognosis.

**Methods:**

A total of 56 patients with CRSwNP reported their self-evaluated olfactory dysfunction score preoperatively and 1 month, 3 months, and 12 months after ESS. Preoperative clinical characteristics, computed tomography (CT) scan, and sinonasal endoscopy examination results were collected before surgery. Additionally, factors that predicted olfactory loss and affected the improvement of olfaction after ESS were evaluated.

**Results:**

Olfactory improvement can be observed 1 month after ESS. A total of 73.2% (41/56) subjects experienced sustained recovery of subjective olfaction with the self-evaluated olfactory dysfunction score improving from 2.04 to 0.64 (*P *< 0.001) after 12 months. The Lund–Mackay scores (*r* = 0.593, *P *< 0.001) and Lund–Kennedy scores (*r* = 0.265, *P *< 0.05) correlated with the preoperative olfactory dysfunction score. Multivariate logistic regression analysis revealed that longer duration of olfactory dysfunction, blood eosinophilia, lower Lund–Mackay scores, and peripheral distribution of CT opacification were risk factors that adversely affected the recovery of olfactory function (*P *< 0.05).

**Conclusion:**

ESS improved self-evaluated olfactory function in patients with CRSwNP. Lund–Mackay scores and Lund–Kennedy scores were correlated with olfactory function prior to surgery, while a longer course of the disease, higher blood eosinophilia, lower Lund–Mackay scores, and peripheral distribution of CT opacification were risk factors for poor olfactory prognosis.

## Introduction

Chronic rhinosinusitis with nasal polyps (CRSwNP) is a chronic sinonasal inflammatory condition with a prevalence of 2.7% (1). It is commonly associated with great quality of life impairment and health care spending (1). Additionally, a diminished sense of smell is one of the predominant dysfunctions present in CRSwNP that may result in patients not being able to identify dangerous situations (such as gas leaks, smoke, or spoiled foods) and often contributes to the development of a depressive disorder (2).

With subjective or objective olfactory tests such as “Sniffin’ Sticks,” several studies (2–4) have demonstrated that endoscopic sinus surgery (ESS) can improve olfaction in patients with CRSwNP, especially those who did not achieve a clinically meaningful recovery after appropriate medical therapy (5). However, the improvement rate varies greatly (25%–100%) (6). On the other hand, olfaction is not always constant and stable in the daily life of patients with CRSwNP. Dynamic changes in olfaction are associated with frequent day-to-day changes in nasal airflow. Because olfactory examinations such as “Sniffin” sticks’ only represent patients’ olfactory level at the time the test is performed, an overall subjective judgment of olfaction by patients over time is also needed to reflect dynamic changes in olfaction as they impact patients’ quality of life.

Identifying risk factors of olfactory dysfunction and postoperative olfactory recovery are essential for guiding physicians and patients regarding outcomes that can be achieved by ESS. Litvack et al. suggested that age, nasal polyposis, smoking, and asthma were associated with olfactory disorders (7). Pade et al. demonstrated that the presence of polyposis was a significant predictor of post-ESS olfactory improvement, while age failed to predict the outcome (4). A prospective study further reported that olfactory cleft opacification and CT scan score were predictive factors for post-ESS olfactory improvement (8).

Beyond these clinical characteristics, eosinophils also play a prominent role in CRSwNP (9, 10). Inflammatory products such as eosinophil-derived neurotoxin (EDN) can damage the olfactory epithelium and result in the apoptosis of olfactory neurons. Stevens et al. suggested that smell loss may be induced by T2 inflammation (11), and Pause et al. demonstrated that ethmoid bulla eosinophilia is associated with olfactory impairment in patients with CRSwNP (12), but the pieces of evidence that elucidate the correlation between eosinophilia and olfactory impairment are still limited, and the role eosinophilia plays in the post-ESS olfactory improvement has not been determined.

The purpose of this study was to analyze the overall subjective olfactory outcomes of ESS using prospectively collected data. Furthermore, we evaluated preoperative factors that predicted subjective olfactory dysfunction and risk factors that were associated with the recovery of subjective olfaction after ESS to lend support for improving diagnosis, optimizing surgical case selection, and counseling patients appropriately.

## Methods

### Subjects

This study was approved by the Medical Ethics Committee of Qilu Hospital of Shandong University. A total of 56 subjects aged 24–63 years who satisfied the diagnostic criteria for CRSwNP established by the European Position Paper on Rhinosinusitis and Nasal Polyps (EPOS2012) (13) and failed medical treatment were recruited from patients referred to the Department of Otolaryngology, Qilu Hospital Shandong University for functional endoscopic sinus surgery. Patients were excluded if they had a history of head trauma, neurologic tumor and neurologic surgery, central nervous system diseases, psychosis, endocrine system diseases, and congenital anosmia.

### Demographics and Comorbidities

Patients’ data including demographics, medical history, medical record information, preoperative risk factors, preoperative medications, previous sinus surgery, and intraoperative and postoperative data were collected from both the patients and the medical records (Table 1). Blood samples were obtained to determine the circulating absolute eosinophil count prior to surgery.

**Table 1:** Characteristics of study patients with CRSwNP (*n* = 56).

Variable	CRSwNP (*n* = 56)
Age, mean (SD), years	47.4 (11.1)
Female, No. (%)	15 (26.8)
Duration of olfactory dysfunction, mean (SD), years	4.8 (6.1)
Asthma, No. (%)	5 (8.9)
Allergic rhinitis, No. (%)	4 (7.1)
Previous sinus surgery, No. (%)	17 (30.4)
Circulating eosinophil count, mean (SD), cells/μl	350.2 (241.7)
Lund–Mackay CT score, mean (SD)	15.8 (5.0)
Lund–Kennedy endoscopy score, mean (SD)	7.3 (2.7)

*SD, standard deviation; N, sample size; CT, computed tomography*.

### Measures of CRSwNP Severity

The extent of CRSwNP was evaluated based on computed tomography (CT) findings, endoscopic findings, and patient-reported SNOT-20 preoperatively. Each patient underwent a high-resolution CT scan of the paranasal sinuses and scored with the Lund–Mackay scoring system (range: 0–24) (14) by experienced physicians. Sinonasal endoscopy was performed and quantified using the Lund–Kennedy scoring system (range: 0–20) (15). Based on the different distributions of CT opacification patterns, we further classified the patients as the central type group (with lesions mainly located near the ethmoid sinus and olfactory region) and peripheral type group (with lesions mainly located in the maxillary sinus). A total of 56 participants were asked to complete the SNOT-20 questionnaire (16) that includes 20 questions that rate both sinonasal symptoms and general quality-of-life parameters with ranges for each question from 0 to 5, where 0 defines no problems and 5 defines the highest impairment.

### Subjective Olfactory Evaluation

All subjects were required to evaluate their olfactory dysfunction based on the overall judgment of recent 1 week preoperatively and 1, 3, and 12 months postoperatively with a 0–3 scale, with 0 for normosmic, 1 for mild impairment, 2 for moderate impairment, and 3 for anosmic. The olfactory improvement score was obtained by subtracting the 12 month postoperative score from the preoperative olfactory score. If the score was more than 0, we classified it as an improved group (Imp); otherwise, we defined it as a non-improved group (N-Imp). This overall subjective judgment of olfactory dysfunction by patients based on the recent 1 week reflects the overall olfaction regardless of the dynamic changes during this period.

### Endoscopic Sinus Surgery Intervention

The approach of surgery was tailored to the extent of lesions as defined by symptoms, CT scan, endoscopy examination, and judgment of the clinic physician. Unless there were obvious polyps in the olfactory cleft area, the surgeon tried not to damage the mucosa of the olfactory area. All cases were followed through postoperative therapeutic regimens including daily nasal saline irrigation, mometasone furoate aqueous nasal spray (200 μg bid), and endoscopic debridement. Subjects were followed up for 12 months.

### Statistical Analyses

Statistical analyses were performed using GraphPad Prism 8 (La Jolla, CA, USA) and SPSS version 26.0 (IBM, Armonk, NY, United States). The associations between preoperative olfactory dysfunction and clinical factors were analyzed. Binary logistic regression analysis was constructed to identify characteristics that affected the improvement of subjective olfactory after surgery and univariate screened was performed before regression analysis. A *P-*value of <0.05 was considered statistically significant.

## Results

### Olfactory Improvement Following Endoscopic Sinus Surgery for CRSwNP

**Figure 1:**
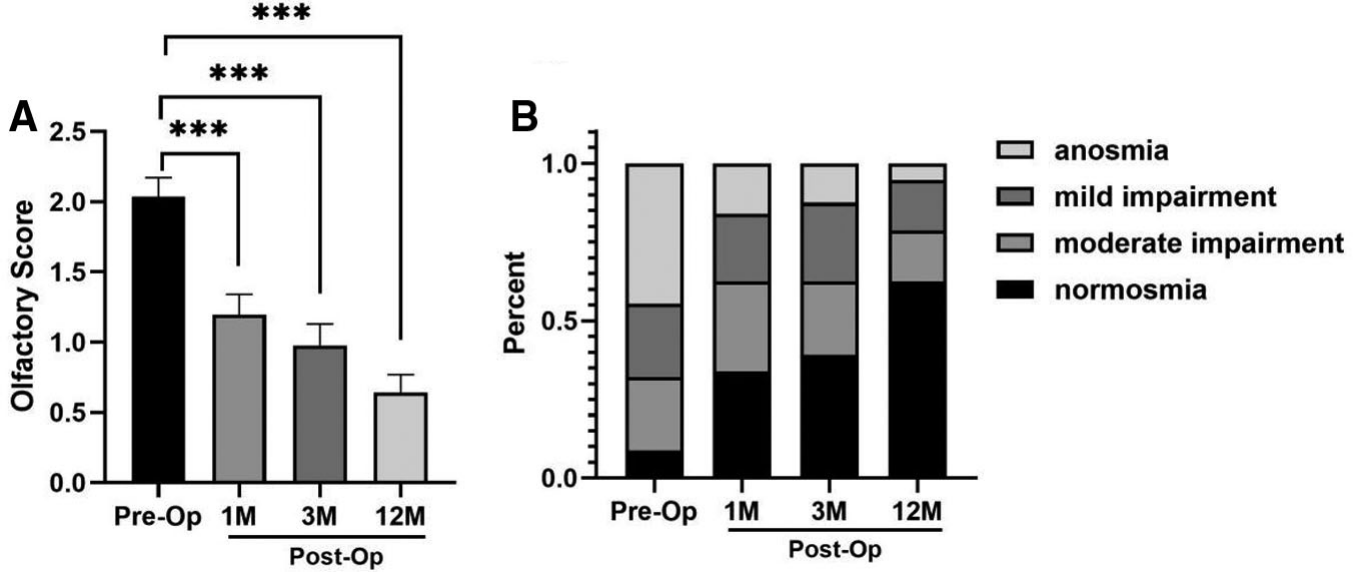
Self-reported olfactory dysfunction score, mean ± SEM (**A**). Distribution variation of the different categories of olfaction preoperatively and 1 month, 3 months, and 12 months after ESS in patients with CRSwNP (**B**) (*n* = 56, ***P < 0.001).

A total of 56 patients with CRSwNP were divided into 4 groups based on their preoperative self-evaluated olfactory dysfunction of which only 8.9% (*n* = 5) presented with normosmia, 23.2% (*n* = 13) had mild impairment, 23.2% (*n* = 13) had moderate impairment, and 44.7% (*n* = 25) had complete anosmia. The mean value of patients’ self-reported olfactory dysfunction score (from 0 = normosmic to 3 = anosmic as described in the Methods section) before surgery was 2.04, and it decreased significantly to 1.20 at 1 month (*P *< 0.001) after surgery. Further improvements in olfaction were observed in the 3rd and 12th months postoperatively as indicated by the olfactory dysfunction score of 0.98 (*P *< 0.001) and 0.64 (*P *< 0.001), respectively (Figure 1A). A total of 41 (73.2%) subjects experienced an improvement in subjective olfaction at 12 months postoperatively, while at the same time, 13 (23.2%) patients reported no change and 2 (3.6%) patients suffered from deterioration.

### Predictors of Olfactory Dysfunction in Patients With CRSwNP

**Figure 2:**
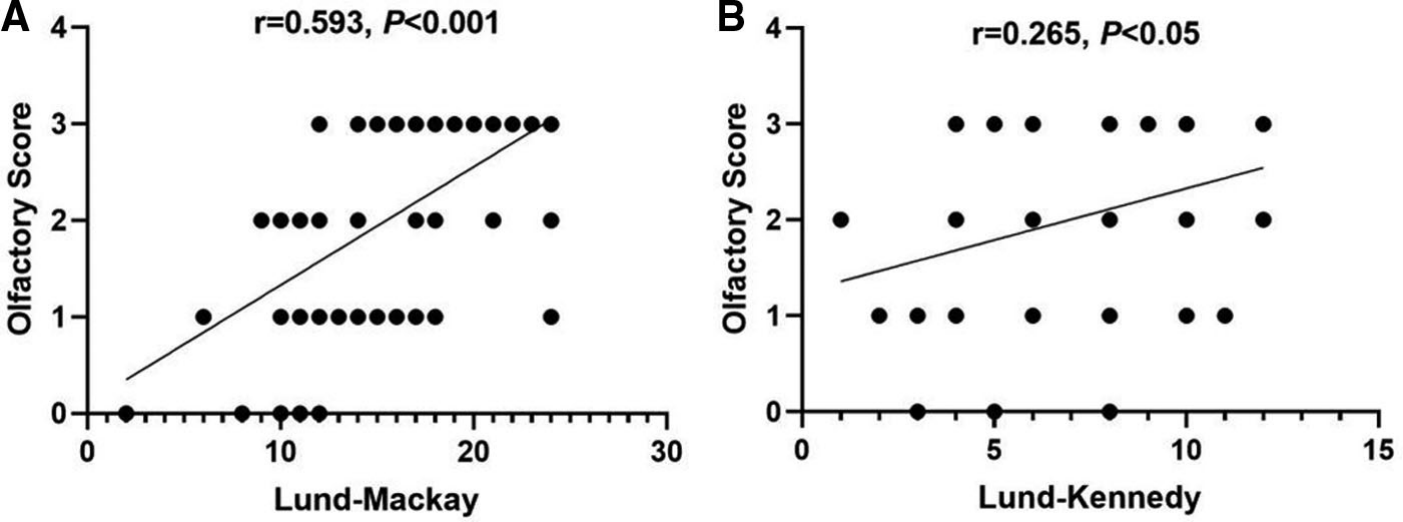
Correlation between olfactory dysfunction score and Lund–Mackay scores (**A**) and Lund–Kennedy scores (**B**). (Some data of different subjects were the same and were not distinguished as separate points in this figure.)

As patients with CRSwNP often adapted to the changes in smell and ignored the olfactory loss, we investigated factors that may reflect subjective olfaction. Presence of asthma, allergic rhinitis, blood absolute eosinophil counts, prior surgery, age, sex, duration of olfactory dysfunction, Lund–MacKay scores, and Lund–Kennedy scores were assessed in this study. Not surprisingly, Lund–Mackay scores (*r* = 0.593, *P *< 0.001) and Lund–Kennedy scores (*r* = 0.265, *P *< 0.05) correlated with the preoperative olfactory dysfunction score (Figure 2). However, neither allergic rhinitis nor asthma was associated with the preoperative olfactory dysfunction score in this cohort (*P *> 0.05). Age, sex, duration of olfactory dysfunction, and blood eosinophil counts did not show any predictive value for preoperative self-reported olfactory dysfunction in these patients either (*P *> 0.05).

### Factors Influencing the Improvement of Olfaction Postoperatively

**Figure 3:**
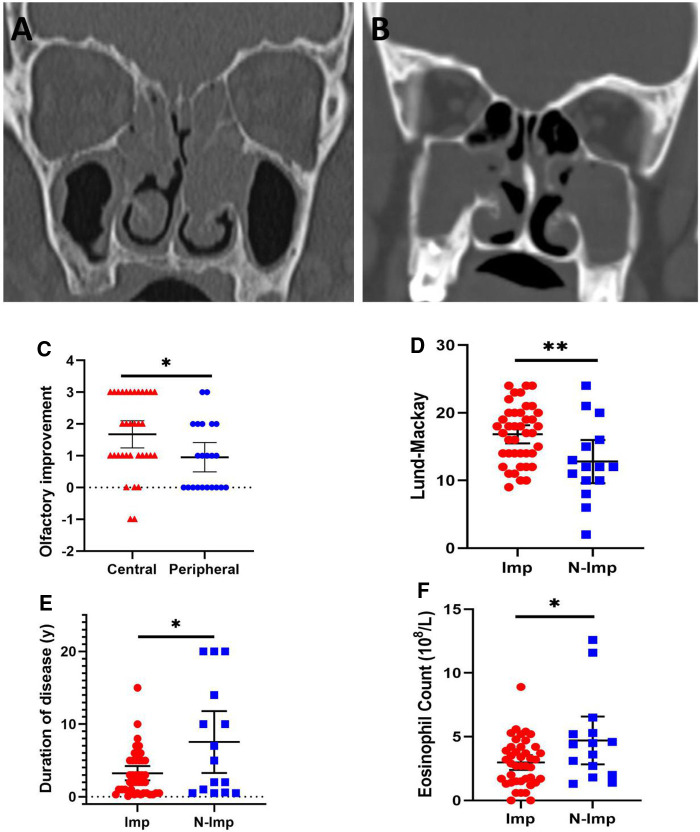
Representative computed tomography imaging of central type (**A**) and peripheral type (**B**) opacification. The central type showed higher olfactory improvement scores compared to the peripheral type (**C**). Nonimproved patients (N-Imp) exhibited lower Lund–Mackay scores (**D**), longer duration of olfactory dysfunction (**E**), and higher circulating eosinophil count (**F**) compared with improved patients (Imp). *P < 0.05, **P < 0.01.

On univariate screening, duration of disease, circulating eosinophil count, Lund–Mackay CT score, Lund–Kennedy endoscopy score, and CT opacification distribution were considered candidate variables for logistic regression modeling (Table 2). Then, multivariate logistic regression analysis examined variables of improvement in the patients’ self-reported olfactory dysfunction after ESS in the predictive model. The duration of olfactory dysfunction, preoperative Lund–Mackay scores, and circulating eosinophil counts showed a predictive value for olfactory prognosis in this model (*P *< 0.05) (Table 3). As olfaction can also be affected by age and gender, so we also included these two variables to build a second logistic regression model (Table 3). The result was the same as the first model. We further demonstrated that different distributions of CT opacification patterns predicted olfactory prognosis. Even though the central type of opacification [with lesions mainly located near the ethmoid sinus and olfactory region (Figure 3A)] presented a worse preoperative olfactory dysfunction score (Table 4, *P *< 0.05) compared with the peripheral type of opacification (with lesions mainly located in the maxillary sinus (Figure 3B)), the central type was associated with higher improvement percentage (Table 5, 85.2% vs. 54.5%, *P *< 0.05) and improvement scores (Figure 3C, *P *< 0.05) compared with the peripheral type. In particular, we analyzed the clinical characteristics of improved and nonimproved patients, demonstrating that the nonimproved group was characterized by a lower Lund–Mackay score (Figure 3D, *P *< 0.01), longer duration of olfactory dysfunction (Figure 3E, *P *< 0.05), and higher circulating eosinophil count (Figure 3F, *P *< 0.05) preoperatively, which were consistent with the multivariate logistic regression model.

**Table 2:** Univariate screening for predictive variables on postoperative olfactory improvement.

Variable	*P*-value
Age, year	0.420
Gender	0.175
Asthma	0.162
Allergic rhinitis	0.935
Previous sinus surgery	0.097
Duration of olfactory dysfunction, year	0.037
Circulating eosinophil count, mean (SD), 10^8^/L	0.022
Lund–Mackay CT score	0.006
Lund–Kennedy endoscopy score	0.040
CT opacification distribution	0.011

**Table 3:** Multivariate logistic regression analysis on postoperative olfactory improvement.

Model	Variable	OR (95% CI)	*P*-value
Model 1			
	Duration of olfactory dysfunction	0.728 (0.572–0.927)	0.010
	Lund–Mackay CT score	1.414 (1.058–1.889)	0.019
	Lund–Kennedy endoscopy score	1.064 (0.735–1.541)	0.742
	Circulating eosinophil count	0.355 (0.169–0.744)	0.006
	CT opacification distribution	78.327 (2.321–2643.555)	0.015
Model 2			
	Age	1.099 (0.987–1.224)	0.086
	Gender	0.135 (0.003–5.813)	0.297
	Duration of olfactory dysfunction	0.653 (0.466–0.913)	0.013
	Lund–Mackay CT score	1.519 (1.079–2.138)	0.017
	Lund–Kennedy endoscopy score	1.061 (0.715–1.573)	0.769
	Circulating eosinophil count	0.319 (0.133–0.768)	0.011
	CT opacification distribution	231.781 (2.980–18,029.800)	0.014

**Table 4:** Comparison of olfactory dysfunction scores.

	Olfactory dysfunction score, mean (SD)			
	Pre-op	1M	3M	12M
Central group	2.26 (0.93)*	1.24 (1.07)	1.08 (1.16)	0.59 (0.92)
Peripheral group	1.68 (1.09)	1.14 (1.13)	0.86 (0.85)	0.73 (0.98)

*SD, standard deviation; Pre-op, pre-operative; M, month*.

* *P < 0.05 compared with peripheral group pre-operatively*.

**Table 5:** Comparison of olfactory improvement.

Group	Count	Improvement	No change	Deterioration	Efficiency (%)
Central	34	39	3	2	85.2
Peripheral	22	12	10	0	54.5

*P < 0.05. The response rate with the central type of opacification was higher than that with the peripheral type. Central type opacification (with lesions mainly located in the ethmoid sinus). Peripheral type opacification (with lesions primarily in the area of non-ethmoid sinuses)*.

## Discussion

Olfactory dysfunction is a common complaint of patients with CRSwNP, which impairs both their general and specific health-related quality of life (17), but the pathophysiology driving olfactory dysfunction is still obscure. Recent evidence suggests it is multifactorial, involving conductive and sensorineural pathways. Obstructive pathology causes decreased airflow to the olfactory cleft and the conductive disorders include nasal polyposis, mucosal edema, and nasal discharge (18). Sensorineural loss implies injury to the olfactory neuroepithelium by chronic inflammation (7).

Accumulating evidence has demonstrated that nasal endoscopic surgery or other comprehensive treatments can significantly improve the olfactory function of patients with CRSwNP based on different methods (subjective or objective) for quantifying olfactory dysfunction (19). Recently, Besser et al. (20) reported that subjective self-ratings of olfaction in patients with CRS can be more accurate than that in patients awaiting septoplasty or septorhinoplasty, indicating the value of subjective olfactory judgment in patients with CRS. Our current observations further provided insight into the therapeutic efficacy of ESS interventions for overall subjective olfactory disorders (not a specific time point), and, specifically, we demonstrated that in 73.2% of CRSwNP patients, olfactory function improved after ESS. An immediate improvement of olfactory function could be observed within 1 month after surgery, a time point at which benefit may reflect the surgical removal of the mechanical obstruction blocking the airflow to the olfactory cleft. Furthermore, removing the inflammatory hyperplastic tissue will promote the self-repair of the nasal mucosa and olfactory epithelium (21), resulting in a further recovery in olfactory function from the 3rd to 12th month after ESS, as was observed in this cohort. This later continuing improvement reflects the greater capacity of nasal steroids to access the olfactory cleft and is consistent with previous research that the olfactory bulb volume increased after ESS (22).

In addition, a small proportion of patients did not get improvement or even had deleterious consequences after ESS. Several points need to be taken into account that could have resulted in a poor outcome: (1) there may have been irreversible damage to the structure of the olfactory area caused by surgery, previous viral infection, or chronic inflammatory stimulation; (2) persistence of poor olfaction from persistent postoperative nasal drainage; and (3) bad tissue repair or insufficient time for olfactory recovery to have occurred.

With chronic obstruction by nasal polyps and damage by inflammation to the olfactory epithelium and nerves, the sense of smell is constantly declining or even permanently lost in CRSwNP, but patients often adapt to changes of smell and ignore the olfactory loss. In this study, we examined clinical characteristics that may help clinicians identify patients with the risk of olfactory dysfunction pre-operatively. In agreement with other studies (23–25), we found that higher Lund–Mackay CT scores and Lund–Kennedy endoscopy scores were correlated with poor olfactory function. However, age, sex, duration of olfactory dysfunction, and absolute eosinophil count did not show any predictive value for preoperative self-reported olfactory dysfunction in these patients (data not shown). Even though there are several proposed mechanisms that could cause olfactory disorder after ESS such as direct injury to the olfactory epithelium, scarring, vascular injury and ischemia, modification of airflow, and effects of a pharmacologic agent (19), patients who had previously undergone ESS did not carry an increased risk of olfactory impairment compared to subjects who had no prior surgery history, suggesting that previous ESS may not be a risk factor for olfactory dysfunction.

As the prevalence of asthma and allergic rhinitis is lower in Chinese compared with Caucasian patients, we only identified five patients with asthma and four patients with allergic rhinitis in this cohort and failed to correlate these comorbid factors with olfactory dysfunction. Even though the “unified airway” theory argues that CRSwNP patients with asthma can be regarded as having systemic inflammation in both the upper and lower respiratory tracts which would influence olfaction (26), asthma and allergic rhinitis may not be universal predictors in the Chinese population due to the low prevalence of these disorders. However, this conclusion needs to be further confirmed in future studies with larger sample sizes.

Eosinophilia is thought to play a prominent role in the pathogenesis of CRS and is regarded as a hallmark and key event of nasal polyps (27), but the correlation between eosinophilia and olfactory disorder has not been fully understood. Hox et al. elucidated that blood eosinophilia correlated with subjective smell reduction (28), Lavin et al. found superior turbinate eosinophilia correlates with an olfactory deficit in chronic rhinosinusitis patients (29), indicating both systemic and local eosinophilia can impair olfaction. We further demonstrated blood eosinophilia predicted poor olfactory prognosis after ESS (*P = *0.006). In this study, the nonimproved group was characterized by higher circulating eosinophil counts compared with the improved group (Figure 3F), which may indicate a persistent Th_2_-type inflammation that could not be improved completely through removing the obstructive and inflammatory tissue by ESS. Activated eosinophils release neurotropic and neurotoxic products which could affect olfactory neuron survival and regeneration (30–32), resulting in poor olfactory prognosis postoperatively. Taken together, these data may indicate that the effect of ESS on improving olfaction through relieving systemic inflammation is still limited. Beyond surgery, those patients with olfactory deficit may further benefit from treatments that reduce systemic inflammation and blood eosinophilia, such as oral corticosteroids.

Consistent with the studies that patients with less than 12 months of sinus burden acquired the greatest benefit after surgery (33) and longer surgical wait times were associated with less improvement in symptoms (34), we further found that patients with a longer course of olfactory loss displayed a worse prognosis of olfaction postoperatively (*P = *0.010). Olfactory epithelium becomes progressively remodeled and replaced by squamous epithelium as nasal inflammation progresses (35). As such, early interventions (at least before these histological changes take place) are needed to obtain greater olfactory improvement.

A prospective study by Vandenhende-Szymanski et al. reported olfactory cleft opacification and CT scan score are predictive factors for post-ESS olfactory improvement (8). In agreement with this study, we found that higher Lund–Mackay scores predicted better olfactory improvement postoperatively (*P = *0.019) and the improved patients exhibited significantly higher Lund–Mackay scores (Figure 3D, *P *< 0.05). Beyond this, we further demonstrated that the distribution of lesions affects the prognosis of the olfactory sense, in which the central type exhibited higher improvement scores (Figure 3C, *P *< 0.05) and improvement percentage (Table 5, 85.2% vs.54.5%, *P *< 0.05) compared with the peripheral type of opacification. The reasonable interpretation of this result is that ESS removed the mechanical obstruction and inflammatory tissue of the olfactory cleft in the central type, while in the peripheral type, the conductive blockage and inflammatory damage may not be accountable for having produced the olfaction disorder.

It is noteworthy that there are several limitations to our investigation. First, subjective olfactory dysfunction ratings were used as the study parameter, which may deviate from objective measures of olfactory function. However, Haxel et al. (21) demonstrated that self-ratings of olfaction correlated well with the Sniffin’ Sticks results. Given that objective smell testing is not widely available, subjective olfactory ratings may also be a useful option. Both subjective olfactory function evaluation and objective olfactory measurement could be used as research parameters in future studies to further our understanding of the efficacy of ESS for olfaction disorder. Second, a repeated olfactory evaluation of different time points by both subjective and objective methods during a specific period may reflect the overall subjective olfactory disorders better, which would both validate the use of subjective scales and confirm the objective improvement in olfaction experienced by patients after sinus surgery. Third, our results may be subject to floor or ceiling effects (36). Finally, the subjective olfactory dysfunction ratings in this study were not validated by other studies, but the SNOT-22 questionnaire rated patients’ subjective olfaction from 0 for no problem to 5 for as bad as it can be, which is similar to our rating system. Future studies on finding a standard subjective olfactory rating system may be needed.

In conclusion, our study demonstrated ESS can effectively improve the subjective olfaction of patients with CRSwNP. Higher Lund–Mackay CT scores and Lund–Kennedy endoscopy scores suggest greater olfactory dysfunction preoperatively. Furthermore, a longer course of the disease, higher blood eosinophilia, lower Lund–Mackay scores, and peripheral distribution of CT opacification are risk factors for poor olfactory prognosis after surgery.

## Data Availability

The raw data supporting the conclusions of this article will be made available by the authors, without undue reservation.
